# The Regulators of Human Endometrial Stromal Cell Decidualization

**DOI:** 10.3390/biom12091275

**Published:** 2022-09-10

**Authors:** Hiromi Murata, Susumu Tanaka, Hidetaka Okada

**Affiliations:** 1 Department of Obstetrics and Gynecology, Kansai Medical University, 2-5-1 Shinmachi, Osaka 573-1010, Japan; 2 Department of Nutrition Science, Faculty of Nursing and Nutrition, University of Nagasaki, Siebold, 1-1-1 Manabino, Nagayo-cho, Nishi-Sonogi-gun, Nagasaki 851-2195, Japan

**Keywords:** human endometrial stromal cells, decidualization, endometrium, progesterone

## Abstract

Several factors are important for implantation and subsequent placentation in the endometrium, including immunity, angiogenesis, extracellular matrix, glucose metabolism, reactive oxidative stress, and hormones. The involvement or abnormality of these factors can impair canonical decidualization. Unusual decidualization can lead to perinatal complications, such as disruption of trophoblast invasion. Drastic changes in the morphology and function of human endometrial stromal cells (hESCs) are important for decidualization of the human endometrium; hESCs are used to induce optimal morphological and functional decidualization in vitro because they contain estrogen and progesterone receptors. In this review, we will focus on the studies that have been conducted on hESC decidualization, including the results from our laboratory.

## 1. Introduction

The human endometrium differentiates into the decidua following the increased secretion of progesterone from the ovarian corpus luteum after ovulation [1]. An embryo that reaches the uterine lumen during the implantation window successfully adheres to the endometrial epithelium, invades and penetrates the decidua, and forms chorionic villi, resulting in embryo implantation [2]. After implantation, trophoblasts alter and remodel maternal spiral arteries, resulting in placentation. The invasion of the decidua by the embryo is a crucial step in the placentation process, and control of trophoblast invasion is one of the most essential functions of the decidua [3]. Notably, spontaneous endometrial decidualization occurs only in species that establish the placenta, with the trophoblasts invading the maternal tissues [4]. Therefore, it is important to recognize that animal models are generally inappropriate for endometrial studies.

When embryo implantation is not achieved, corpus luteum regression causes a decrease in progesterone levels and shedding of the decidua [5]. This physiological phenomenon is termed menstruation. After menstruation, endometrial repair, proliferation, and differentiation occur in the human endometrium in preparation for another embryo implantation [6]. During the female reproductive life span, 450 cycles of ovarian sex steroid hormones repeat the menstrual cycle leading to decidualization of the endometrium [5,7,8]. There are two dominant phases in the endometrial cycle: the proliferative phase—in which re-epithelialization, stromal proliferation, and angiogenesis occur with increased levels of estradiol (E2) from menstruation to ovulation—and the secretory phase—including the receptive period, in which the endometrium transforms into a form suitable for implantation with increased levels of progesterone after ovulation. The stromal cells, epithelial cells, vascular endothelial cells, and leukocytes that comprise the endometrium are differentiated by rising progesterone and intracellular cyclic adenosine monophosphate (cAMP) levels after ovulation, with the cell–cell interactions promoting decidualization. This decidual reaction, even during pregnancy, functions as an essential role in the pregnancy establishment.

**Figure 1:**
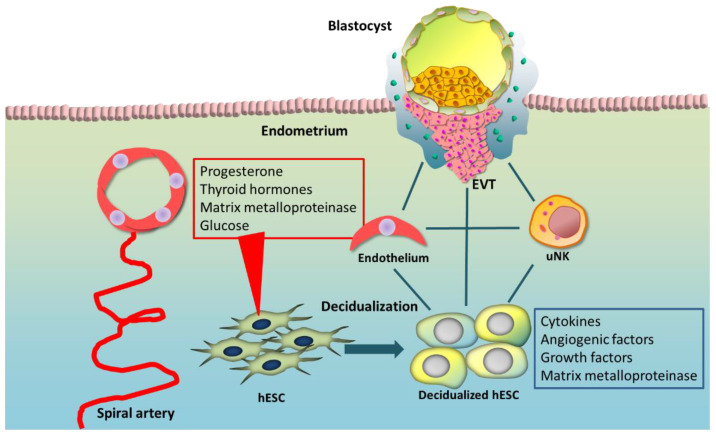
Decidualization of human endometrial stromal cells (hESCs). hESC decidualization is essential for successful reproduction. During the secretory phase, hESCs interact with endometrial cells including vascular endothelial cells, decidual natural killer (dNK) cells representing local immune cells, and blastocyst. Progesterone, thyroid hormones, matrix metalloproteinases, and glucose affect the regulatory mechanism during hESCs decidualization.

The decidualization process is characterized by changes in human endometrial stromal cell (hESC) morphology, increase in uterine gland secretion, elevation of endometrial-specific uterine natural killer (uNK) cells, and rebuilding of blood vessels to supply the growing embryo with gases and nutrients from maternal blood [6,9]. In contrast, the decidualized hESCs become the major cell type in the decidua [10]. During decidualization, hESCs with fibroblast-like shapes differentiate into epithelial-like cells in the secretory phase and in pregnancy [11] (Figure 1). Decidualized hESCs are defined as epithelial-like cells with enlarged round nuclei, increased number of nucleoli, dense-core secretory granules located around membrane boundary, accumulation of lipid droplets and glycogen in cytoplasm, and enlarged rough endoplasmic reticulum with the Golgi complex [1,12]. With an increase in progesterone levels before and after ovulation, the endometrium begins to decidualize. In the mid-secretory period, the stromal cells around the arteries (Figure 1), which are included in the upper two-thirds region, initiate these morphological changes, and implantation is possible from approximately six days after ovulation, with or without a conceptus [1]. Pregnancy consists of a complex process in which a series of discrete events occur, with decidualization being the earliest and significant of these processes.

Because it is not possible to study hESC decidualization directly in vivo for ethical reasons, cell culture models have been developed to study decidualization in vitro. hESCs can be purified from human endometrial stromal fibroblasts of women with normal menstrual cycles and have been cultured and studied in vitro [13,14,15]. Treatment of these cultured hESCs with progesterone for 12 days causes morphological differentiation and expression of decidualization markers such as prolactin (PRL) and insulin-like growth factor binding protein 1 (IGFBP1) [16,17]. On the other hand, intracellular cAMP levels have been reported to increase during decidualization several days after progestin administration [18]. Factors known to be able to induce accumulation of cAMP in hESC cells include ovarian hormones, relaxin, corticotropin-releasing factor, and prostaglandin E2 [19,20]. cAMP has become known as an inducer of decidualization and is involved in PRL and IGFBP1 expression [1]. Indeed, upon removal of cAMP stimulation, decidualized hESCs revert back to an undifferentiated phenotype and no longer express PRL or IGFBP1 [18]. In hESCs, combination with cAMP and progestin alters the elevated PRL expression to a greater extent than cAMP-alone treatment. However, several genes in hESCs are upregulated only by progestin and not by cAMP [18,21,22], suggesting a direct pathway via a genomic and/or non-genomic progesterone-stimulated pathway and an indirect pathway via cAMP to hESC decidualization. That is, during hESC decidualization, progestin-induced activation of locally expressed factors and the cAMP second messenger pathway integrates hormonal inputs and confers cell specificity on progesterone action [18]. Various transcription factors critical for hESC decidualization are either repressed or acutely upregulated rather than upregulated for an extended period of time [23]. These results suggest that the decidualization step works in the direction of releasing the brakes on an already working system rather than turning the system from completely off to completely on. Therefore, the energy required to “switch on” decidualization is low, which is hypothesized to be the result of a large amount of energy being expended to effectively inhibit decidualization at a time of low progesterone levels [23].

In this review, we will introduce the results of our work to date and the significance of decidualization and the factors that regulate decidualization, including these transcription factors.

## 2. Functions of Decidualization

The human endometrium is differentiated into decidua by the action of progesterone secreted from the ovarian corpus luteum after ovulation. The endometrium is composed of stromal cells, glandular cells, immune cells, and vascular endothelial cells, and the interaction between these maternal cells and the embryo promotes decidualization from embryonic implantation to placentation. The establishment of pregnancy depends on the interaction between the embryo and the maternal decidua during the process of placentation. Upon arrival in the uterus, the embryo attaches to the endometrial glandular epithelium, invades the decidua, and is nourished by the endometrium until the uteroplacental circulation is established. The embryo differentiates into an embryoblast that differentiates into individual tissues and a trophectoderm that is involved in placental formation. Among the trophoblasts, extravillous trophoblast (EVT) cells first invade the dense decidua layer. The trophoblast is remodeled into a highly permeable spiral artery by invading the uterine spiral artery and replacing the maternal vascular endothelial cells. Thus, gas exchange and nutrient transport, which are essential for the life support and growth of the embryo, are provided by the placenta. The invasion of the embryo into the decidua is an essential process for placentation, and the control of invasion is one of the important functions of the decidua [3]. Furthermore, the decidua plays an important role in immune tolerance to avoid rejection of embryos that carry some of the paternal DNA, a contradictory feature that promotes the ability to protect the mother by eliminating pathogenic microorganisms [24].

Decidual transformation of the endometrium mainly occurs in hESCs to accommodate extravillous trophoblasts (EVTs) that invade during implantation [1]. Defects in decidualization can result in alterations in immune cells, including uNK [25,26] and endometrial stem cells [27,28], and in endometrial breakdown and a variety of pregnancy disorders, including infertility, recurrent miscarriages, and utero-placental disorders [1,29,30,31]. Additionally, abnormalities in decidualization have been implicated in diseases such as endometriosis, in which impaired decidualization leads to ectopic uterine tissue growth. Chronic deciduitis, a chronic inflammation of the decidua, has been linked with premature birth [32].

It has recently been shown that decidualization is accompanied by cellular senescence [33,34,35,36]. Although senescence was initially thought to be an intrinsic anti-tumor barrier that prevents the proliferation of damaged cells [37], it was later suggested that senescence-associated secretory phenotype (SASP) produced and secreted by senescent cells promotes tumor development in the pre-neoplastic periphery and has an active role in cancer progression [38,39]. Furthermore, accumulation of senescent cells in tissues has been demonstrated to mediate dysfunction and the progression of various pathologies [40,41,42]. In the endometrium, aging changes in the hESC secretome, similar in many ways to SASP, were shown to precede implantation failure [43]. Based on DNA methylation analysis, aging hESCs are implicated in reduced endometrial plasticity and in failure to implant [27]. Implantation failure was associated with increased levels of aging hESCs during the proliferative phase of the menstrual cycle [44]. In addition, a small number of primary cultured hESCs from patients without endometrial lesions scheduled for in vitro fertilization demonstrated a strong correlation between senescence marker levels and inhibition of decidualization, decreased invasive capacity, and implantation failure rates [45]. IL15 secreted from decidual hESCs activates uNK cells, and activated-uNK cells might eliminate senescent decidual hESCs [33]. In the decidualization process, the balance between senescence and differentiation needs to be appropriately maintained, and disruption of this balance may lead to abnormal decidualization [34,46] and subsequent pre-term birth [35,36,47]. Senescent hESCs were found to reinforcing dysfunction of the stromal compartment via secretome changes [45]. Application of senomorphic agents to suppress the senescence phenotype reduced the negative effects on decidualization and implantation of senescent hESCs [45]. These suggest that anti-aging agents can prevent the adverse effects of the senescent hESCs against decidualization of “healthy” surrounding hESCs and on embryo implantation.

On the other hand, application of the anti-aging agent rapamycin reduced PRL and IGFBP1, major secretory proteins in decidual hESCs, and expression in hESCs, as well as impairing decidualization [33]. In addition, another anti-aging agent, resveratrol, was also shown to have an anti-decidualization effect that reduced the expression of PRL and IGFBP1 [48]. Clinical trials have found that continuous resveratrol supplementation also causes reduced implantation rates [49]. However, these results are strictly related to the phases of the menstrual cycle [50], suggesting that the addition of anti-aging agents to the implantation window can adversely affect embryo implantation by disrupting the appearance of cell subpopulations that emerge during hESC decidualization [33,49]. Methods that do not affect embryo implantation, such as supplementation of anti-aging agents only during the proliferative phase, should also be kept in mind. Therefore, administration during the proliferative phase of the menstrual cycle has the potential to decrease the production of SASP and may be a promising strategy to further prepare the endometrium for embryo implantation.

PRL and IGFBP1 are the major secretory proteins in decidual hESCs (Figure 1) and, therefore, have been used as decidualization markers. The proposed roles of these proteins at the decidual–placental interface include stimulation of trophoblast growing and penetration, evasion from immunological refusal, survival regulation for the uNK cell, and promotion of neovascularization [1,29,51] (Figure 1). There are three subsets of decidualized hESCs at the maternal–fetal border: decidual stromal cells (dS) 1, 2, and 3 [3]. In the secretory phase, the decidua—the superficial layer in two thirds of the endometrium—splits into two layers: a compact outer layer (stratum compactum) and an intermediate layer (stratum spongiosum). dS2 and dS3, both of which highly express IGFBP1 and PRL, are present in the stratum compacta, whereas dS1, which does not express IGFBP1 and PRL, is present in the stratum spongiosa. It was also found that EVT first invades the dense layer of the decidua, where dS2 and dS3, which highly express the immune checkpoint molecules galectin 9 (*LGALS9*) and C-type lectin domain family 2 member D, are present. These may suppress the immune response and facilitate EVT invasion.

## 3. Regulators for Decidualization

### 3.1. Ovarian Steroid Hormones

Increased E2 and progesterone cause decidualization [52]. These hormonal fluctuations during the menstrual cycle promote the differentiation required for implantation [53]. E2 regulates the progesterone receptor (PGR) transcription and adjusts the endometrial microenvironment to accommodate progesterone during the secretory phase [54,55]. Progesterone establishes and maintains the pregnancy with increased levels in the secretory phase and maintenance of increased levels throughout pregnancy [52]. Infertility and recurrent miscarriages have been associated with post-ovulatory progesterone deficiency [56].

Progesterone activates PGRs, resulting in the homeostasis of endometrium for pregnancy preparation. In humans, high expression of PGRs in hESCs is observed from the secretory phase to pregnancy, but that expression after ovulation is reduced in endometrial epitheliums. In PGR-knockout mice, endometrial decidualization was incomplete, resulting in multifaceted reproductive abnormalities [57]. Thus, progesterone is an essential signal for decidualization and a prerequisite for successful implantation. In other words, progesterone acts as the master regulator of pregnancy.

### 3.2. Transcriptional Regulators Induced by Ovarian Steroid Hormones in Decidualization

Within the cell, E2 and progesterone function through nuclear transcription factors (TFs), estrogen receptors (ESRs), and PGRs [58]. In the presence of these hormonal regulators, hESCs differentiate to build the endometrial distinctive networks in gene expression that influence the menstrual cycle and gestation. Various TFs are involved in hESCs decidualization, in concert with or downstream of ESR and PGR. CCAAT/enhancer binding protein β (CEBPB) promotes the differentiation of hESCs [59]. CEBPB is essential for decidualization, which does not occur in the absence of CEBPB in murine uteri [60]. Homeobox A10 (HOXA10) is expressed primarily in the endometrium and is significantly elevated during the implantation window [61]. Low levels of HOXA10 expression are accompanied by altered expressions of the endometrial receptor marker, β3-integrin, and empty spiracles Homeobox 2 [62]. HOXA10 regulates the transcription of FK506-binding protein 4, which enhances progestin-mediated transcription [63]. Forkhead box protein O1 (FOXO1) competes with PGR to control the expression of the gene subset involved in decidualization [64]. A negative regulator for decidualization, Krueppel-like factor 12, shows direct binding to the FOXO1 upstream region and inhibits FOXO1 in hESCs, resulting in impaired decidualization and implantation failure [65]. 

We found that FOXO1 and its phosphorylation transcriptionally regulate *LGALS9*, a protein secreted by hESCs that is involved in uNK differentiation [66]. Furthermore, FOXO1 may not be involved in the expression of *interleukin 15* (*IL15*) [67]. There are several sites of FOXO1 phosphorylation, and each site determines whether the transcriptional activity of FOXO1 is turned on or off; therefore, detailed mapping of the FOXO1 phosphorylation sites associated with decidualization is necessary. Heart and neural crest derivatives expressed 2 (HAND2) functions as a critical role in endometrial receptivity by suppressing E2 signal [68,69,70,71,72,73]. In hESC decidualization, progestin increases HAND2, and knockdown by siRNA inhibits morphological differentiation and decreases the levels of decidua-specific proteins such as FOXO1 [74]. The crucial HAND2 role in the *IL15* transcription in hESCs has also been demonstrated [67]. However, HAND2 alone does not fully explain the transcriptional regulation mechanism of *IL15*, and it is necessary to explore the mechanism from various perspectives, such as interactions with HAND2. HAND2 phosphorylation has also been reported [75]; therefore, the role of phosphorylated HAND2 in hESC decidualization requires further investigation.

Thyroid hormones (THs) are known to be important factors for the regulation of ovulation, fertilization, implantation, and early fetal development [76]. Overt hypothyroidism must be treated because of its adverse effects against the fetus and obstetrics. In contrast, the pros and cons of treatment during pregnancy for subclinical hypothyroidism, a mild hypothyroidism with elevated thyrotropin and standard value in free TH, remain controversial [77]. An association of subclinical hypothyroidism during pregnancy with an increased miscarriage and preterm delivery rate and a decrease in the child’s intelligence quotient has been established [77]. A randomized controlled trial of levothyroxine (LT_4_) replacement therapy for subclinical hypothyroidism showed improved delivery and decreased miscarriage rates [78]. The American Thyroid Association 2017 guidelines highly recommend LT_4_ treatment for women with subclinical hypothyroidism who undergo intracytoplasmic sperm injection or in vitro fertilization [79].

**Figure 2:**
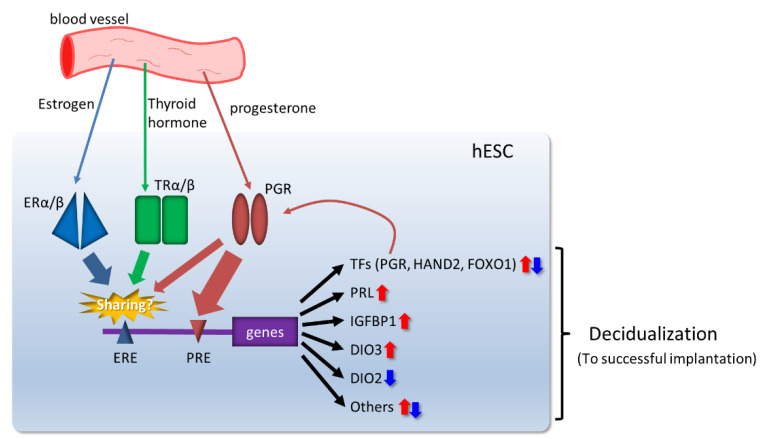
Involvement of thyroid hormones (THs) in hESC decidualization. ERE: estrogen response element; PRE: progesterone response element; TFs: transcription factors; PGR: progesterone receptor; TR: TH receptor; ER: estrogen receptor.

After TH is converted from thyroxine (T_4_) to its active form, tri-iodothyronine (T_3_), various genes are regulated in various parts of the body [80]. TH-mediated signaling pathways depend on the expression of cell- and tissue-specific multiple TH receptor (TR) isoforms and iodothyronine deiodinase (DIO) [81]. Recently, we found that hESCs treated with a combination of ovarian steroid hormones and LT_4_ show elevations in decidual markers PRL and IGFBP1 and significant increases in a number of transcription factors [82]. We also verified that TH-induced decidualization in hESCs was inhibited by simultaneous TRα and TRβ silencing. Furthermore, we observed that PGRs increased during TH-induced decidualization [82]. While binding to their receptors, TH and reproductive hormones activate common signaling pathways and build a complex and reciprocal network owing to sharing of the consensus sequences and their protein similar structure [83,84] (Figure 2). PGR signaling, regulated by TH, is considered one of the key players in decidualization.

We also found that DIO3, which converts T_4_ to an inactive form of reverse T_3_, is upregulated, and DIO2, which converts T_4_ to T_3_, is downregulated during hESC decidualization in the presence of TH [82]. TH functions even against trophoblasts and regulates endocrine functions, such as steroid synthesis, in early pregnancy [85]. Strong evidence in the uterus of rat models show that DIO3 is strongly transcribed to protect against early fetal exposure to maternal TH [86]. TH during gestation is thought to affect both the endometrium and fetus, controlling the implantation process.

### 3.3. Immune Response in Endometrial Microenvironment

The decidua has an essential function in helping the semi-allogeneic feto–placental unit evade the maternal immunological reaction as well as generating an immune-tolerant environment in the placenta [87,88]. Properly decidualized hESCs are important for the development of maternal immune tolerance and acquisition of distinctive roles involved in the perception, choice, and admittance of the homologous embryo [89]. 

uNK cells localized to the decidua basalis, which are called decidual natural killer (dNK) cells, make up seventy percent of endometrial leukocytes throughout the secretory phase to early pregnancy. Although dNK cells are considered a distinct population from their counterparts in blood, according to phenotypic characterization and functional analysis during the first trimester in humans, tissue-resident natural killer (trNK) cells and circulating natural killer (cNK) cells are known to be a source of uNK in mice [90,91]. Pregnant uterine tissue contains cNK cells and trNK cells as well as innate lymphoid cells, which can rapidly respond to virally infected or altered cells, collectively called uNKs [92,93]. On the other hand, these three possibilities for the origin of human dNK cells are known: (1) peripheral blood CD16-NK cells differentiated into dNK cells in the endometrial microenvironment, (2) direct differentiation from hematopoietic progenitor cells in the endometrium, and (3) peripheral CD16+ dNK cells differentiated under the influence of TGFβ and other factors [94].

dNK cells are less cytotoxic and play important roles in placentation and maintenance of pregnancy, including immune tolerance in the decidua, promotion of angiogenesis, remodeling of spiral arteries, infiltration of the trophoblast, and elimination of senescent decidualized hESCs linked to recurrent pregnancy loss [28,33,95,96,97]. dNK cells exhibit tissue-resident markers such as CD9, CD69, and CD49a, as well as adhesion molecules such as CD9, CD62L, and α-1 integrin, which may aid in their accumulation [98,99]. It also contains more lysozymes such as perforin [100]. Furthermore, dNK cells express high levels of killer cell immunoglobulin-like receptors and CD94/natural killer group 2 member A, which can bind to different types of HLA on trophoblast cells, suggesting that dNK cells regulate trophoblast cells [24,101].

B cells, T cells, mast cells, macrophages, dendritic cells, and neutrophils may be involved in immune tolerance and embryo implantation besides the predominant uNK cells [24,102]. dNKs obtained from the first trimester decidua of a healthy woman reportedly express the antimicrobial peptide granulysin. dNKs contain more granulysin than peripheral NK cells and supply granulysin to the placenta constitutively [103]. dNKs strongly express granulysin and selectively transfer it to EVT through nanotubes to kill the intracellular Listeria monocytogenes but not trophoblasts [95]. 

On the other hand, the uNK cell phenotype of nonpregnant endometrium has not yet been fully investigated. A study comparing nonpregnant and pregnant endometrial CD56+ cells found 450 differentially expressed genes, with 70% overexpressed in the nonpregnant uNK cell subset [104]. Therefore, far from being inactive, nonpregnant uNK cells may play an important role in implantation and early placenta [104].

The influx, activation, and survival of uNKs in the human endometrium has been linked to IL15 [33,105]. In mice deficient in *IL15*, decidualization induces inappropriate differentiation, resulting in loss of mature dNKs. When these mice are treated with IL15, dNK cell populations are maintained [106]. The uNK cells secrete neovascularization factors, such as angiopoietins (ANGPTs) and vascular endothelial cell growth factors (VEGFs), and interferon-γ (IFNG) [8] to regulate endometrial vascular remodeling. However, the participation of dNKs in angiogenesis remains controversial, as Vento-Tormo et al. found no evidence that VEGFA or IFNG are substantially expressed by dNKs in vivo [3].

While IL15 secreted by hESCs via progesterone stimulation is non-dispensable in the differentiation of uNK cells, endometrial leukocytes including uNK cells do not express PGR in humans [107]. In the human endometrium, during the secretory phase, high *IL15* transcription is observed [67,108]. During the secretory phase of the endometrium, histological analysis has revealed elevated levels of *IL15* in many hESCs [67]. Progestin-induced decidualization causes increased IL15 secretion by primary cultured hESCs [109]. Recent single-cell analysis has defined the interaction between IL15 in decidualized hESC and its receptors in dNK as a critical event for placentation [3]. Furthermore, IL15 is the lifeline of uNKs.

After implantation is completed, placental EVTs invade the decidua and migrate towards the placental spiral artery [110]. EVTs invade the decidua compacta first, where dS2 and dS3 express high levels of *LGALS9* [3,66]. LGALS9 binds to β-galactoside [111] and is secreted by the ESCs; it also interacts with hepatitis A virus cellular receptor 2 (HAVCR2), an inhibitory receptor on a subset of uNK and EVTs [3,24]. Interaction between LGALS9 and HAVCR2 causes suppression of the inflammatory responses in the decidua [112,113,114,115,116]. Decreased levels of HAVCR2-positive uNK are observed in miscarriages in human and in abortion-prone mice [117]. LGALS9-HAVCR2 signaling may trigger EVT responses, such as progesterone [118] and profilin-1 secretion, as feedback regulators to promote hESCs decidualization [119].

### 3.4. Matrix Metalloproteinases (MMPs)

Human implantation and placentation require deep invasion of the endometrium by EVTs [110]. The decidual stromal cells produce extracellular matrix (ECM) and interact with the invading EVT to restrict the degree of EVT invasion [51]. Adherens and gap junctions exist between decidual stromal cells, which may be arranged for EVT invasion. Decidualized stromal cells produce several ECMs, including fibronectin, type IV collagen, laminin, heparin sulfate proteoglycans, and decorin [8,120].

To invade the decidua, EVTs require ECM remodeling after proteolytic degradation. MMPs, which are produced by EVT, have a prominent function in regulation of the EVT invasive capacity by degrading ECM components [51,121]. The decidua simultaneously produces tissue inhibitors of metalloproteinases (TIMPs), which antagonize MMPs and limit EVT invasion, protecting the endometrium. Hence, the decidua is believed to function as a biochemical and physical barrier that restricts EVT entry.

**Figure 3:**
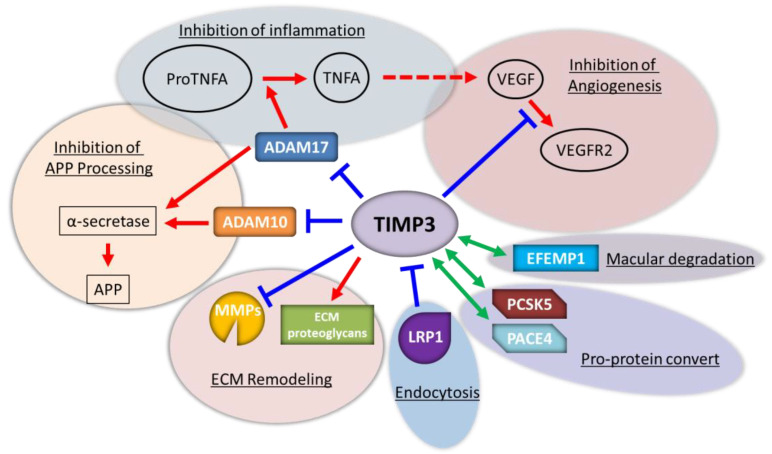
The versatile roles of tissue inhibitors of metalloproteinases (TIMP) 3. Red, blue, and green lines indicate activation, inhibition, and interaction, respectively.

However, changes in MMPs in the decidualized stromal cells have also been reported [122]. As expected, many MMPs were suppressed; however, contrary to expectations, MMP15 and MMP19 were upregulated. MMP15 is the sole human placental membrane-type MMP expressed throughout early pregnancy and has been postulated to assist cytotrophoblast invasion in concert with cytotrophoblasts. PGR regulates MMP19 during decidualization [123]. MMP19 induced by PGR is reduced in animals with low pregnancy rates and is thought to maintain vascular stability during decidualization [124,125]. In addition to MMP involvement in invasion and vascular remodeling, which are assumed functions of MMPs in the endometrium, involvement of hESCs in cell migration has also been suggested. Recent reports have shown that decidualization of hESCs is accompanied by an increase in cell migration [126]. Indeed, MMP inhibition in human cardiomyocyte progenitor cells has been reported to inhibit cell mobility [127]. Additionally, genome-wide siRNA screening found that several MMPs were modulators of decidualization [23]. The increased mobility of hESCs may allow for the placement of sufficient uNKs and blood vessels at the right time and place, as well as control of trophoblast invasion. TIMP3 has MMP-independent functions and interacts with various factors without MMPs [122] (Figure 3). For example, TIMP3 inhibits VEGF [128], regulates disintegrin and metalloproteinase domain-containing protein (ADAM) 17 activity post-translationally [129], and regulates the amyloid precursor protein by decreasing the surface levels of ADAM10 [130,131]. Therefore, TIMP3 inhibits the migration and growth of vasoendothelials and increases vascular permeability; it is also increased during progestin-induced hESC decidualization [22]. 

### 3.5. Endometrial Vascular Remodeling and Maturation

Rapid vascular growth occurs with increasing E2 levels in the proliferative phase, and vascular maturation is controlled by progesterone in the secretory phase [132,133]. hESC decidualization commences during the secretory phase underneath the luminal epithelium and around the terminal spiral arteries [134]. The endometrial vasculature facilitates decidualization by providing a stable transport of endocrine hormones, metabolites, oxygen, and immune cells to hESCs. Meanwhile, decidualized hESCs further promote angiogenesis and maturation of the endometrial vasculature [8]. Pericytes and vascular smooth muscle cells as perivascular supporting cells (also called mural cells) are the blood vessel components and maintain vascular stability [135]. Paracrine control by ANGPTs from mural cells to the endothelial Tie2 receptor plays an essential function in the differentiation of perivascular mesenchymal cells into pericytes and the maintenance of tight interactions between endothelial and mural cells [135]. Moreover, for those in the early decidua between the maternal and fetal interface, significant ligand–receptor pair-mediated specific interactions among endothelial and perivascular cells, EVTs, decidualized hESCs, and immune cells have been demonstrated [3].

Endometrial vascular remodeling is controlled by E2 and progesterone, which act indirectly via essential endometrial regulators, including VEGF, ANGPTs, and fibroblast growth factors (FGFs) [8,136]. The ANGPT family of secreted growth factors consists of ANGPT1, ANGPT2, and the orthologs—ANGPT3 and ANGPT4 [135]. All secreted ANGPTs are ligands of the Tie2 receptor. ANGPT1 matures blood vessels by recruiting pericytes and stabilizing their attachments. ANGPT2 antagonizes ANGPT1, thereby promoting neovascularization with VEGF [137]. Using primary cultured hESCs and an immortalized hESC cell line KC02-44D, our group unexpectedly identified that cigarette smoking extract increases VEGF in hESCs [138,139]. The empirically known adverse effects of smoking are thought to occur by inappropriately altering endometrial angiogenesis. We also found that progesterone suppresses ANGPT2 production, maintains ANGPT1 levels in hESCs, and reduces the ratio of ANGPT2/ANGPT1 [140]. While FGF9 acts as an intermediary of ANGPT2, progesterone-induced HAND2 may suppress FGF9 and contribute to vascular maturation during hESC decidualization [141]. A recent study found that the endometrial vascular lumen is enlarged in the secretory phase rather than in the proliferative phase [142]. In contrast, there are fewer blood vessels with narrower vascular lumens in the decidua of women suffering from miscarriage compared to those of healthy pregnant women. [142]. Vascular remodeling and maturation, controlled by sex hormones, play an essential function in successful pregnancy.

### 3.6. Glucose Metabolism

During the peri-implantation period, embryonic development and endometrial differentiation require considerable energy. Therefore, there is a high demand for the glucose that enters the glycolytic system to produce energy molecules such as ATP and NADH [143]. Thus, glucose is essential for progesterone-induced hESC decidualization [144,145]. In decidualized hESCs, glucose uptake is increased and low-glucose conditions induce PRL suppression [145].

However, since the embryo and endometrium cannot synthesize glucose, it must be delivered to them by a glucose transporter (GLUT). Seven GLUTs are expressed in the human endometrium, among which GLUT1 is the most important [146,147,148]; GLUT1 is regulated by insulin signal activation and endometrial epigenetic regulation [145,149,150]. GLUT1 protein expression is increased in decidualized hESCs, and *GLUT1* silencing reduces *PRL* and *IGFBP1* [144,146]. The presence of INSR, ISR1, and IRS2 in the endometrium is now known [145], and IRS2, which is directly regulated by the progesterone receptor, is also reportedly responsible for GLUT1 expression and plasma membrane localization in the human decidua [150]. Furthermore, epigenetic H3K27ac modification upregulates *GLUT1* [149].

The immature vascular structure of the endometrium during implantation suggests that it is a hypoxic environment. Increased expression of HIF1α and HIF2α, which are elevated under hypoxic conditions, was found in the mouse and human uterus during embryonic implantation [151,152]. Metabolome analysis revealed that hESCs cultured under hypoxic conditions suppressed oxidative metabolism and enhanced glycolytic metabolism [153]. Under hypoxic conditions, we observed increased GLUT1 expression levels and HIF1α inhibition induced the suppression of GLUT1 transcription. Furthermore, GLUT1 knockdown suppresses glucose uptake by 90% [153], confirming the importance of GLUT1 in the endometrium.

## 4. Conclusions

Several factors, including ovarian steroid hormones, oxidative stress, thyroid hormones, and glucose, influencing genomic or non-genomic transcript regulation are involved in hESC decidualization. The proper action of these factors results in optimal endometrial differentiation and harmonious interactions among hESCs, EVTs, the endothelium, and immune cells. Recent excellent reports found 4238 novel genetic and chemical modulators of decidualization by using immortalized hESCs with yellow fluorescent protein under the control of the prolactin promoter and a genome-wide siRNA screening [23]. In addition to G-protein receptors and the ubiquitin–proteasome system, which have not been previously recognized but are expected to be involved to some extent, 87 unexpected olfactory receptors were identified as modulators of hESC decidualization. Thirty-six homeodomain transcription factors, which are known to be involved in ontogeny, are also identified. Even more interesting is the large number of cXXorfXs, DKFZPs, FLJs, KIAAs, LOCs, and MGC genes whose functions are unknown, and further analysis of these may lead to the discovery of novel functions in decidualization and treatments of pregnancy disorders. Additionally, numerous molecular biological studies have suggested that the endometrium is affected by the endocrine system, glucose metabolism, and lifestyle. Finally, endometrial preconception care is important for the success of reproduction because hESC decidualization begins before the embryo reaches the superficial endometrium in humans.

## Data Availability

No new data were created or analyzed in this study. Data sharing is not applicable to this article.
